# Pre-Clinical Case Competition to Assess Confidence in Responding to Select Out-Of-Hospital Medical Emergencies

**DOI:** 10.5070/M5.52198

**Published:** 2026-01-31

**Authors:** Harrison Fillmore, Thomas Heisler, Marissa Nadeau, Emmagene Worley, Lauren Titone, Tiffany Murano, Jimmy Truong

**Affiliations:** * Columbia University Vagelos College of Physicians and Surgeons, New York, NY; ^ Columbia University Irving Medical Center, Department of Emergency Medicine, New York, NY

## Abstract

**Audience:**

This session is intended for first- and second-year medical students, or any pre-clinical medical students.

**Introduction:**

Preclinical learners build skills and confidence when they practice first responder tasks early in training, and simulation helps them to perform basic procedures better.^1^,^2^ A small group case competition uses a game format to reinforce rapid assessment, clear team roles, and closed-loop communication.^3^,^4^

Out-of-hospital cardiac arrest affects hundreds of thousands of people in the United States each year, and survival to discharge is low. Bystander cardiopulmonary resuscitation (CPR) and early use of an automated external defibrillator (AED) improve outcomes.^5^,^6^ Anaphylaxis is a time-sensitive emergency seen in the community and in the emergency department (ED). Epinephrine is the first-line treatment, and delay to administration worsens outcomes.^7^,^8^ Opioid overdose remains a major cause of preventable death. Early recognition, respiratory support, and Naloxone administration are key steps.^9^

**Educational Objectives:**

By the end of this activity, learners will be able to:

1. Demonstrate the application of skills in real-life first responder scenarios, including suspected opioid overdose, cardiac arrest, and anaphylaxis.
2. Apply knowledge of scene safety and the role of the first responder in various situations.
3. Assess the challenges while applying the skills necessary for collaborative work within a medical team.

**Educational Methods:**

A competition combining simulation-based and team-based learning reinforced first responder skills among first- and second-year medical students. Attending physicians evaluated simulated out-of-hospital emergency scenarios using a detailed rubric. This method was chosen to engage learners in a lower-stakes (but still simulated high-pressure) assessment of their skills where undifferentiated patients challenge recall and application in new, previously unknown scenarios.

This format was chosen to keep preclinical students active and focused while they practice time-critical first responder skills. Simulation allows decision-making, hands-on actions, and immediate feedback in a safe setting. Team-based learning mirrors ED teamwork by assigning clear roles, prompting closed-loop communication, and requiring shared problem-solving. Station design supports repeated practice and brief debriefs, which builds retention and confidence for early learners. Faculty-scored rubrics provide observable, standardized performance measures and make feedback specific and actionable.

**Research Methods:**

Pre- and post-intervention surveys assessed the effectiveness of the case competition featuring three simulation scenarios in improving students’ confidence in managing emergency situations.

**Results:**

The competition increased participants’ confidence in responding to various scenarios, particularly opioid overdoses. Most participants expressed high likelihood of participating in future competitions. Ten preclinical students completed pre- and post-session surveys. Confidence increased across the cohort. For the opioid overdose scenario, “complete confidence” rose from 1/10 (10%) pre- to 6/10 (60%) post. Confidence in cardiac arrest and anaphylaxis also trended upward. Likelihood to respond to a public emergency changed minimally. Interest in future events was high, with 6/10 (60%) extremely likely and 4/10 (40%) somewhat likely to participate again.

**Discussion:**

The competition successfully increased confidence and fostered collaboration but faced recruitment challenges due to its optional nature and scheduling. Future implementations could include more advanced learners.

**Topics:**

First aid, first responder, competition, pre-clinical.

## USER GUIDE

**Table:** 

List of Resources:	
Abstract	29
User Guide	31
Small Groups Learning Materials	36
Appendix A: Faculty Brief Case 1: Anaphylaxis	39
Appendix B: Faculty Brief Case 2: Opioid Overdose	43
Appendix C: Faculty Brief Case 3: CPR – AED – ECG Recognition	48
Appendix D: Debrief Pearls	53
Appendix E: EM Interest Group Case Competition Pre-Survey	55
Appendix F: EM Interest Group Case Competition Post-Survey	57

**Learner Audience:**

This case is directed to medical students.

**Time Required for Implementation:**

Small Groups: 2hrs 30min from start to finish: 30 minutes for set up, 5 minutes for introductions, division into teams, and explaining flow; 20 minutes at each station with 10-minute breaks in between for grading, evaluation, and shuffling (2 hours total)

Debriefing: 10 minutes

**Recommended Number of Learners Per Instructor:**

4–6

**Topics:**

Opioid overdose, anaphylaxis, cardiac arrest, CPR.

**Objectives:**

By the end of this activity, learners will be able to:

1. Demonstrate the application of skills in real-life first responder scenarios, including suspected
1. opioid overdose, cardiac arrest, and anaphylaxis.
2. Apply knowledge of scene safety and the role of the first responder in various situations.
3. Assess the challenges while applying the skills necessary for collaborative work within a medical team.

### Linked objectives and methods

Early exposure to clinical experiences is an important aspect of preclinical medical education. Allowing students to practice clinical skills early in their careers has been shown to improve confidence and patient care skills in medical students with limited prior clinical experience. Even in students with prior clinical experience, early exposure to practical skills training increased medical knowledge and professionalism.^1^ In addition to providing students with practical skills, early clinical education is important in professional identity formation. Previous educational studies have shown that first aid courses were important in preparing preclinical students for basic medical emergencies, as well as helping facilitate the transition to becoming a medical professional.^3^

Inclusion of first aid education in the medical school curriculum has received positive student feedback and demonstrated the effectiveness of a first responder course through significant improvements in students’ knowledge and practical skills, increased confidence levels, and enhanced performance in Objective Structured Clinical Examinations (OSCEs).^10^,^11^ While first aid courses have been shown to be successful in teaching students to respond to out-of-hospital medical emergencies, student feedback has demonstrated the need to reinforce this content without being repetitive.^10^ Game-based learning modules have been shown to increase student engagement and learning value more than traditional lectures.^12^ As such, we aim to demonstrate the educational value of a case competition in which first-year and second-year medical students work together to treat medical emergencies in a simulated out-of-hospital setting to reinforce first-aid skills taught earlier in the preclinical curriculum.

We utilized a group competition format based on a pre-clinical first responder curriculum. This is an educational method increasingly studied and utilized.^13^ Groups, consisting of learners at varying levels, work together to identify and treat undifferentiated patients in out-of-hospital emergencies. This focuses students to recall the skills they were presented in the pre-clinical first responder course. Simulation-based learning (SBL) has been shown to improve clinical skills and confidence in managing acute medical situations, with studies demonstrating its effectiveness in developing clinical performance and decision-making abilities.^1^,^2^,^3^,^10^, ^14^ Team-based learning, which emphasizes collaborative learning and teamwork, has also been recognized as an effective educational strategy in medical education.^4^

The case competition utilized a combination of SBL and TBL to reinforce critical first responder skills among first and second-year medical students; it was held in classroom settings with portable low-fidelity simulation props (such as airway and CPR-training mannequins). These participants had previously completed a first responder course, ensuring a baseline level of knowledge and skills pertinent to addressing out-of-hospital emergency scenarios. Although they were not specifically briefed on the course content immediately before the case competition, the pre-session surveys used to evaluate the session did allude to the content they would encounter in the scenarios. Ten first and second-year medical students voluntarily participated in the case competition. They engaged in simulated scenarios designed to mimic real-life emergency situations, including suspected opioid overdose, cardiac arrest, and anaphylaxis. During each case, attending physicians observed students and used a detailed grading rubric to score their performance based on the successful execution of specific tasks critical to patient survival. These tasks included identification and management of emergency conditions, accurate and timely administration of appropriate interventions, and effective communication and teamwork. No specific preparation was required of faculty facilitators besides the training they had already undergone as board-certified emergency medicine physicians. Given that the subject matter is well within the expertise of board-certified emergency medicine physicians, facilitation (including course content expertise, group evaluation, session debrief, and individualized education) was thought to be well within the skillset of those chosen to facilitate. Participants were incentivized with gift-card awards for the members of the winning team.

### Achieving Goals/Objectives

**Educational Methods:** The selected format of a case competition was chosen to foster an interactive, engaging, and high-stakes learning environment. This method combines Simulation-Based Learning (SBL) and Team-Based Learning (TBL), which have been shown to improve clinical skills and confidence in managing acute medical situations.

**Simulation-Based Learning (SBL):** SBL allows students to engage in realistic medical scenarios, providing hands-on experience in managing emergencies. It promotes active learning, immediate application of knowledge, and critical thinking. The competition format enhances the perceived stakes, motivating students to perform at their best while allowing for immediate feedback from instructors, which is crucial for reinforcing learning and boosting confidence.

**Team-Based Learning (TBL):** TBL emphasizes collaborative learning and effective communication within a team. Medical emergencies often require coordinated efforts, and this format allows students to experience and overcome the challenges of working within a medical team. Through team-based activities, students learn to rely on each other’s strengths, improving their ability to function effectively in real-life medical settings.

### Conceptual Framework

**Experiential Learning Theory:** This theory underpins the design of the case competition. According to Kolb’s Experiential Learning Theory, learning is a process whereby knowledge is created through the transformation of experience. The competition provides:

- **Concrete Experience:** Students engage in realistic medical scenarios, gaining hands-on experience in managing emergencies.
- **Reflective Observation:** After each scenario, students reflect on their actions, discussing what went well and areas for improvement.
- **Abstract Conceptualization:** Students conceptualize their learning by analyzing the scenarios and discussing best practices and theoretical underpinnings with peers and instructors.
- **Active Experimentation:** Students apply their new understanding in subsequent scenarios, refining their skills through practice.

**Situated Cognition:** The case competition situates learning in the context of realistic scenarios. This approach ensures that knowledge is learned and applied in an authentic context, making it more likely to be retained and transferred to real-life situations.

By utilizing a combination of SBL and TBL within the framework of Experiential Learning Theory and Situated Cognition, the case competition format effectively meets the educational objectives. It enhances students’ confidence, provides practical skills application, and reinforces the importance of scene safety and teamwork in emergency scenarios.

### Recommended pre-reading for facilitator

- **Opioid Overdose**
  ○ How to respond to an opioid overdose. U.S. Department of Health and Human Services. HHS.gov. Accessed August 26, 2024. https://www.hhs.gov/opioids/treatment/overdose-response/index.html
  ○ Veld M, Hirshon J. Basic cardiopulmonary resuscitation. In: Tintinalli JE, Ma O, Yealy DM, et al, eds. *Tintinalli’s Emergency Medicine: A Comprehensive Study Guide*. 9^th^ ed. McGraw-Hill Education; 2020.
- **Anaphylaxis**
  ○ Allergic reaction/anaphylaxis: causes, symptoms, how to help. American Red Cross. RedCross.org. Accessed August 26, 2024.
  ○ https://www.redcross.org/take-a-class/resources/learn-first-aid/allergic-reaction-anaphylaxis
  ○ Rowe BH, Grunau B. Allergy and anaphylaxis. In: Tintinalli JE, Ma O, Yealy DM, et al, eds. *Tintinalli’s Emergency Medicine: A Comprehensive Study Guide*. 9^th^ ed. McGraw-Hill Education; 2020.
- **Cardiac Arrest**
  ○ Treatment of cardiac arrest. American Heart Association. Heart.org. https://www.heart.org/en/health-topics/cardiac-arrest/emergency-treatment-of-cardiac-arrest.
  ○ Veld M, Hirshon J. Basic cardiopulmonary resuscitation. In: Tintinalli JE, Ma O, Yealy DM, et al, eds. *Tintinalli’s Emergency Medicine: A Comprehensive Study Guide*. 9^th^ ed. McGraw-Hill Education; 2020.

### Learner responsible content (LRC)

Students must have completed a First Responder–level course (or school-approved equivalent) prior to this event covering: CPR/AED, basic airway management (BVM), recognition/management of anaphylaxis, and opioid overdose with Naloxone. No additional pre-session didactics are provided.

### Small group application exercise (sGAE)

See the following attached materials for this small group exercise

- Appendix A: Faculty Brief Case 1: Anaphylaxis
- Appendix B: Faculty Brief Case 2: Opioid Overdose
- Appendix C: Faculty Brief Case 3: CPR – AED – ECG Recognition
- Appendix D: Debrief Pearls
- Appendix E: EM Interest Group Case Competition Pre-Survey
- Appendix F: EM Interest Group Case Competition Post-Survey

### Results and Tips for Successful Implementation

Students completed a pre- and post-intervention survey employed to evaluate the efficacy of the case competition in assessing confidence levels of first and second-year medical students in managing emergency scenarios. All survey responses were de-identified to protect participant confidentiality and were securely stored in databases approved by the institution in compliance with data security protocols. All participants who had previously completed the mandatory First Responder course were recruited voluntarily. The intervention consisted of three low-fidelity simulation scenarios: opioid overdose, cardiac arrest, and anaphylaxis. Each scenario was designed to mimic real-life out-of-hospital emergencies and provide hands-on experience. The scenarios were created by a team of Emergency Medicine faculty to ensure accuracy and educational value. Each scenario was limited to 20 minutes, with a total of three cases conducted during the competition.

#### Participants

The research population included first- and second-year medical students who had previously completed a mandatory pre-clinical first responder course. Invitations to participate were sent via email and class announcements. Participants were encouraged to join based on their interest in improving their emergency response skills.

#### Simulation Scenarios

In the first semester of their first year, all students take a first responder course that highlights skills to respond to emergencies such as opioid overdose, allergic reactions, and cardiac arrest. The scenarios for this study were derived from that course material. During each case simulation, groups were evaluated using a rubric based on tasks such as identifying and managing the emergency condition, administering appropriate interventions, and demonstrating effective communication and teamwork. Each station was allotted 20 minutes, providing ample time for the students to engage with the scenario and receive feedback. The competition format allowed for dynamic interaction and immediate feedback from the observers, enhancing the learning experience. The stations were held in the simulation center in available classrooms.

#### Surveys

To measure the impact of the case competition on students’ confidence levels, pre- and post-intervention surveys with 5-point Likert-scale questions (1=least confident/least likely, 5=most confident/most likely). The pre-competition survey included 10 questions about the students’ confidence in responding to potential anaphylaxis, opioid overdose, cardiac arrest, and their likelihood of responding to a medical emergency in public. The post-competition survey repeated these confidence assessments and additionally included questions about the student’s enjoyment of the competition, likelihood of attending future events, and any feedback for the organizers.

Survey results were analyzed to determine changes in confidence levels before and after the competition. Descriptive statistics summarized participant demographics and baseline characteristics. Likert-scale items were coded from 1 to 5 and summarized using medians and interquartile ranges (IQRs), given the small sample size and ordinal nature of the data. This methodology draws on established practices in simulation-based learning and team-based learning and provides a robust framework for evaluating educational interventions in medical training.^1^,^2^,^3^,^10^,^14^ Ethical considerations included ensuring data confidentiality and securing approval from the institutional review board (IRB), which reviewed the study and found it to be not human subjects research and therefore exempt from IRB approval processes.

Responding to real-life first responder medical scenarios requires confidence, spontaneous action, and teamwork skills. To empower participants to learn about medical emergencies (and how they themselves may respond in a medical emergency), some of the most common scenarios were presented to groups with minimal direction and prior information. The format challenged them to be spontaneous, creative, and to recall diagnoses and treatments presented to them several months prior. We hypothesized that this recall and practice, combined with the higher-stakes competitive environment, would optimize the practical skills gained and confidence built in this educational exercise.

Participants’ confidence in their own ability to respond to the medical emergencies presented in the competition unanimously increased across all scenarios. On a 5-point Likert scale, median confidence in responding to anaphylaxis increased from 3.5 (IQR 3–5) pre-competition to 4.5 (IQR 4–5) post-competition. Confidence in responding to opioid overdose increased from 4.0 (IQR 3–4) to 5.0 (IQR 4–5) and confidence in responding to cardiac arrest increased from 3.5 (IQR 3–4) to 4.5 (IQR 4–5) with n=10 for all three scenarios. This effect was most pronounced in the opioid overdose scenario, wherein only one participant expressed “complete confidence” in responding before the competition, whereas after the competition, six out of ten participants expressed “complete confidence” with the increase in median confidence from 4.0 to 5.0.

The case competition was overall a success, as measured by the survey results and qualitative evaluations of the competition by its participants and judges; it was well-received and increased collaboration, camaraderie, and teaching between doctors and students alike. When compared with more traditional educational formats such as lectures with written or simulated assessments, the competition format may confer additional benefits specifically in building confidence of participants in their skills and teamwork. One takeaway that the authors noticed is the challenge in recruiting participants for the case competition. Two reasons why recruitment was not optimal were that the competition was optional and not within normally scheduled curricular time. While the authors attribute this difficulty to the rigorous demands of medical school, there may be another reason why such an educational format may not be as desirable to some learners: for example, some learners are uncomfortable in competitive environments and may not feel comfortable sharing their suggestions and thought processes in such an environment. Another limitation of the format is variable inter-rater reliability, and different degrees of leniency were likely present among the evaluators, but the use of a specific grading rubric mitigated these effects.

While this case competition focused on the education of medical students (and therefore tested basic medical concepts and widely established protocols), future implementations of this case competition may seek to test retention of these topics in third- and fourth-year medical students or trainees further in their careers. To maximize the ability of this session to increase the confidence of participants responding to medical emergencies, solidify the long-term retention of a first-responder curriculum, and expose participants to the challenges and rewards of working as part of a coordinated team, we recommend the following: allow time between the first-responder curriculum and the implementation of this session; after the first pass of information, minimize the amount of further preparation students undergo prior to the session; design teams so that there are members of many different years and classes.

## Figures and Tables

**Table:** 

Scheduling Matrix							
Location	Agenda	Start	End	Duration	3 Stations		
**Main Lobby**	**Intro/Setup**	4:45 PM	5:20 PM	0:35	**Anaphylaxis (Room 1)**	**Opioid OD (Room 2)**	**CPR/AED (Room 3)**
**Rooms 1,2,3**	**Session 1**	5:20 PM	5:50 PM	0:30	Team 1	Team 2	Team 3
**Rooms 1,2,3**	**Session 2**	5:50 PM	6:20 PM	0:30	Team 2	Team 3	Team 1
**Rooms 1,2,3**	**Session 3**	6:20 PM	6:50 PM	0:30	Team 3	Team 1	Team 2
**Main Lobby**	**Break**	6:50 PM	7:05 PM	0:15			
**Main Lobby**	**Closing**	7:05 PM	7:20 PM	0:10			
**Total**				2:30			

**Figure:**
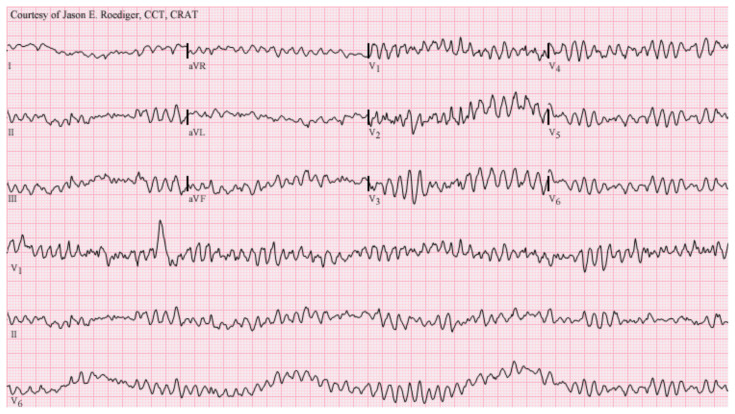
Jer5150. Ventricular Fibrillation. In: Wikimedia Commons. https://commons.wikimedia.org/w/index.php?curid=19669589. 30 May 2012. GNU Free Documentation License, Version 1.2

**Figure:**
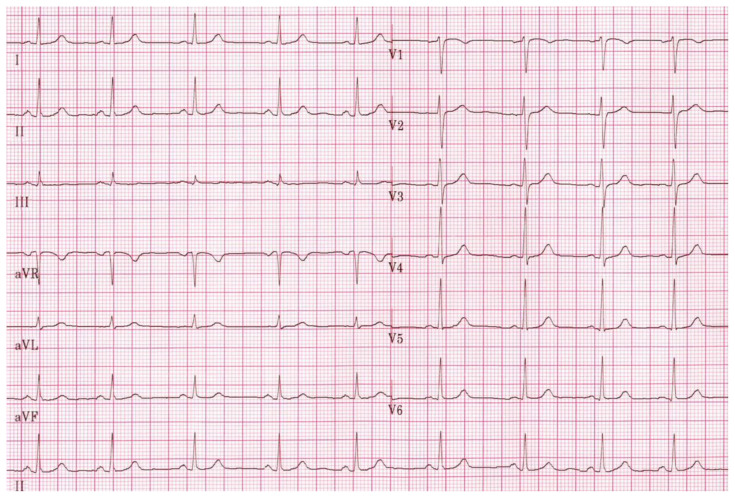
CardioNetworks. Normal sinus rhythm. Wikimedia Commons. https://commons.wikimedia.org/w/index.php?curid=24373409. 25 July 2007, 10:03. Creative Commons Attribution-Share Alike 3.0 Unported.

**Figure:**
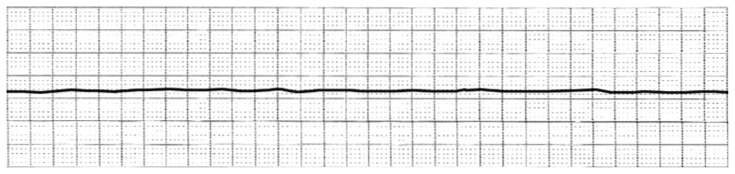
EKG-Ableitung einer Asystolie. Wikimedia Commons. https://commons.wikimedia.org/wiki/File:EKG_Asystole.jpg. 23 November 2006. GNU Free Documentation License, Version 1.2

## SMALL GROUPS LEARNING MATERIALS

### Small Group Application Exercise

#### Format & Grouping

- Total session duration: up to 2 hr and 30 mins
- 4–6 students per group
- Four faculty members (1 per station)
- Location: Simulation Center or any open classroom

#### Run of Show

- Faculty setup: 30 minutes before student start time to review materials and stage equipment
- Student orientation: 5 minutes
- Station rotations
- 15 min. run case + 5-minute immediate debrief (at the station)
- 10 min. transition/grading to next station
- 10 min. end of event debrief with revealing of highest-scoring group
- 15 min. break
- Consider a prize for the highest scoring group, such as gift cards

Please see a mock-up of the scheduling matrix for three stations to help plan your event:

#### Stations

##### Station Setup for All Stations

- **Space & layout:** One clear labeled station sign; manikin/actor centered; table for supplies; timer; binder with relevant case information
- **Staffing & roles:** 1 faculty per station; 4–6 learners per rotation
- **Materials at the table:** Each station should print out the respective appendix to their case, personal protective equipment (PPE), binder with case material (case prompts, ECG or images if applicable), scoring sheet
- **Timing per rotation:** 15-minute case; 5 min debrief; 10 min reset/transition/scoring
- **Reset between groups:** Return props to start positions, replace trainer caps, replace manikin, reset timer, submit scores

###### Station 1: Allergic Reaction/Anaphylaxis (Scene: Bakery)

**Visuals/props:** Doll with facial moulage (puffy eyes/erythema), cookie prop, Epi auto-injector trainer.
**Start state:** Patient seated, pruritic/flush, worsening swelling after eating cookie; no Epi given yet.
**Minimum equipment:** Epi trainer, PPE.
**Instructor notes:** Ensure epinephrine site/hold time is demonstrated; prompt 911/transport plan and brief differential & pathophysiology during debrief.
**Reset:** Reposition doll/props; re-cap Epi trainer; wipe surfaces.

###### Station 2: Opioid Overdose/Naloxone (Scene: Subway Platform)

**Visuals/props:** Manikin supine, intranasal Naloxone trainer.
**Start state:** Unresponsive, slow/shallow or absent respirations, pinpoint pupils (as cue).
**Minimum equipment:** Naloxone trainer, PPE.
**Instructor notes:** Look for recognition of overdose, call for help, airway/ventilation first, then Naloxone; discuss repeat dosing and transport/monitoring.
**Reset:** Re-cap Naloxone trainer; return dummy to start position; wipe BVM/mask if used.

###### Station 3: Cardiac Arrest (CPR-AED-ECG Recognition) (Scene: Gym)

**Visuals/props:** Adult CPR manikin, AED trainer with adult pads, ECG rhythm strips (binder with ≥3 rhythms).
**Start state:** Unresponsive, pulseless; AED closed and pads packaged.
**Minimum equipment:** CPR manikin, AED trainer, rhythm strips for post-case ID.
**Instructor notes:** Emphasize high-quality CPR (rate 100–120, full recoil, minimal pauses), early AED, correct pad placement; after ROSC, have learners identify 3 rhythms from binder.
**Reset:** Replace AED pads on backing, close AED, reset manikin chest recoil counter if present, file rhythm strips.

## Appendix A Faculty Case Brief Case 1: Anaphylaxis

**Visuals/props:** Doll with facial moulage (puffy eyes/erythema), cookie prop, Epi auto-injector trainer.

**Start state:** Patient seated, pruritic/flush, worsening swelling after eating cookie; no Epi given yet.

**Minimum equipment:** Epi trainer, PPE.

**Instructor notes:** Ensure epinephrine site/hold time is demonstrated; prompt 911/transport plan and brief differential & pathophysiology during debrief.

**Reset:** Reposition doll/props; re-cap Epi trainer; wipe surfaces.

**Case Topic: Allergic Reaction/Anaphylaxis**

**Time:** 20 min.

**Goals:** Identify signs and symptoms, provide initial assessment and management of someone with a potential anaphylactic reaction.

**Objectives:**

- Determines the scene is safe
- Calls for help
- Introduces self
- Verbalizes what is happening to person in distress
- Elicits medical history, allergies, and medications
- Verbalizes and assesses
  ○ Airway
  ○ Breathing
  ○ Circulation
  ○ Disability
  ○ Exposure
- Correctly verbalizes a need for an EpiPen
- Administers epinephrine appropriately
- Assess the need for repeated doses
- Instructs that the patient needs to go to the hospital
- Provides differential diagnoses
- Verbalizes dangerous aspects of anaphylaxis

**Scene:** The Bakery

**Props:**

- Doll with Moulage with puffy eyes and face, erythematous hands and face
- Cookie
- EpiPen trainer

**Scenario Set Up:** Patient with pending anaphylactic reaction to nuts after eating a cookie. Participants need to assess the patient and acknowledge the need for EpiPen injection. Participants will need to have patient go to the hospital and provide differential diagnoses and discuss pathophysiology.

**Narrative for facilitator to be read to group:**

You all are sitting at the table enjoying cookies.

As the nanny, you present to the group and scream, “something is wrong!”

You are hysterical and can provide the following information if prompted:

Child presents with puffy face, flushed skin, covered in rash, holding a half-eaten chocolate chip cookie. “She just ate the cookie, and she is not looking well!”

Other information that may be asked:

Person is at the bakery with you (the facilitator) eating a cookie.

**Name:** John Jacob Abel

**Age:** 2

**Gender:** Male

**Medical hx:** None

**Surgery hx:** None

**Medications:** None

**Dosage (if asked):** None

**Allergies:** Nut Allergy

**Physical Exam (if not indicated, normal):**

General: Able to talk to you, breathing fast
No vital signs. If a manual measurement is asked for:
Pulse: 110 bpm; Respiratory Rate: 30 breaths per minute
HEENT: Eyes and face appear puffy and red, tongue is not swollen
Skin and musculoskeletal (MSK): Upper extremity and chest are red, rash that blanches, and is itchy
Lungs: Hears wheezes if asked if they hear anything, tachypneic

**Differential Diagnosis:**

Anaphylaxis, Allergic Reaction, Foreign Body Airway Obstruction

**Assessment of Knowledge Questions:**

What are absolute contraindications to usage of EpiPen?

ANSWER: None

How often can you repeat doses?

ANSWER: Every 5–15 minutes

What is the pathophysiology behind an allergic reaction?

ANSWER: Degranulation of mast cells causing histamine release

How does epinephrine work to treat anaphylaxis?

ANSWER: counters histamine by constricting blood vessels and relaxing the airways

**TEAM: _________________________________**

**SCORER: _______________________________**

**Table:**

Scoring Rubric (25 points max)	Points Possible	Points Awarded
*Scene Size Up (verbally evaluates scene safety)*	1	
*Requests additional assistance (calls for 911, call for help)*	1	
*Introduces self and assignment of roles (delegates roles)*	1	
*Verbalizes what is happening to person in distress (what is happening, differential?)*	1	
*Elicits medical history, allergies, and medications (asks these questions)*	1	
*Verbalizes and assesses* - *Airway* - *Breathing (Look, listen, and feel)* - *Circulation* - *Disability* - *Exposure*	5	
*Correctly verbalizes a need for an EpiPen*	5	
*Administers epinephrine appropriately (demonstrates on doll)*	3	
*Assesses the need for repeated doses*	1	
*Correctly instructs patient to follow up at the hospital*	1	
*Assessment of Knowledge Questions*	4	
*Team organization and style*	1	

## Appendix B Faculty Case Brief Case 2: Opioid Overdose

**Visuals/props:** Manikin supine, intranasal Naloxone trainer.

**Start state:** Unresponsive, slow/shallow or absent respirations, pinpoint pupils (as cue).

**Minimum equipment:** Naloxone trainer, PPE.

**Instructor notes:** Look for recognition of overdose, call for help, airway/ventilation first, then Naloxone; discuss repeat dosing and transport/monitoring.

**Reset:** Re-cap Naloxone trainer; return dummy to start position; wipe BVM/mask if used.

**Case Topic: Opioid Overdose**

**Time:** 20 min.

**Goals:** Identify signs and symptoms, provide initial assessment and management of someone with a potential opioid overdose.

### Objectives

- Determines the scene is safe
- Requests for help
- Introduces self
- Verbalizes what is happening to person in distress
- Attempts to wake the patient up (shake, rub sternum, yell)
- Elicits medical history, allergies, and medications
- Verbalizes and assesses
  ○ Airway
  ○ Breathing
  ○ Circulation (check carotid pulse, examine skin, lips, nails, for cyanosis)
  ○ Disability
  ○ Exposure
- Examine pupils for size
- Elicit any pertinent history if applicable
- Identify signs and symptoms in someone with a potential opioid overdose
- Determine the need for administration of Naloxone
- Lay patient flat on back and administer Naloxone
- Assess the need for repeated dosages
- Acknowledge need for transport to hospital

**Scene:** Subway Station

**Props:**

- Scrub Dummy
- Intranasal Narcan

**Scenario Set Up:** Patient with potential opioid overdose.

Learners able to identify the signs of opioid overdose.

Learners administer Naloxone.

Learners determine the need for repeat dosing and transport to hospital.

**Narrative for facilitator to be read to group:**

“You enter the subway, waiting for the train when you notice someone lying face down across the bench. The person is not moving.”

As the facilitator, after 10 seconds of shaking, you turn and say, “I don’t think he is breathing.”

If they ask for History - no Hx is available.

Other information that may be asked:

**Name:** John/Sally

**Age:** Appears middle-aged

**Medical hx:** Unable to be obtained

**Surgery hx:** Unable to be obtained

**Medications:** Unable to be obtained

**Dosage (if asked):** Unable to be obtained

**Allergies:** Unable to be obtained

**Physical Exam (if not indicated, normal):**

General: Not responsive to you
No vital signs. If a manual measurement is asked for:
Pulse: 50 bpm; Respiratory Rate: 6 breaths per minute
HEENT: Small pupils, no signs of trauma, blue lips
Lungs: His chest moves to what appears to be a breath
Circulation: Slow pulse at around 50 bpm
Skin: Pale skin, blue lips

**Differential Diagnosis:**

Opioid Overdose, hypoglycemia, intoxication, head trauma

**Assessment of Knowledge Questions:**

What is Naloxone (Narcan)?

ANSWER: Opioid Receptor Antagonist
Narcan binds stronger to opioid receptors than opioids do
Does NOT eliminate opioids from the body

How is Naloxone (Narcan) commonly administered out of hospital?

ANSWER: Nasally is most common.
Also available subcutaneously, intramuscularly, or intravenously.

Where are opioid receptors found?

ANSWER: Opioid receptors are found throughout the body on nerves that control pain but also pupils, consciousness, and most importantly, muscles of respiration.

**TEAM: _______________________________**

**SCORER: _______________________________**

**Table:**

Scoring Rubric (25 points max)	Points Possible	Points Awarded
*Scene Size Up (verbally evaluates scene safety)*	1	
*Requests additional EMS assistance*	1	
*Introduces self and assignment of roles*	1	
*Verbalizes what is happening to person in distress*	1	
*Attempt to wake the patient up (shake, rub sternum, yell)*	1	
*Elicits medical history, allergies, and medications*	1	
*Verbalizes and assesses* - *Airway* - *Breathing (look, listen, and feel)* - *Circulation* - *Disability* - *Exposure*	3	
*Examine pupils for size*	3	
*Identify signs and symptoms in someone with a potential opioid overdose (ask this question)*	6	
*Determine the need for administration of Naloxone*	1	
*Lay patient flat on back and administer Naloxone*	1	
*What is on your differential diagnosis?*	1	
*Acknowledge need for transport to hospital*	1	
*Assessment of Knowledge Questions*	3	

## Appendix C Faculty Case Brief Case 3: CPR-AED-ECG Recognition

**Visuals/props:** Adult CPR manikin, AED trainer with adult pads, ECG rhythm strips (binder with ≥3 rhythms)

**Start state:** Unresponsive, pulseless; AED closed and pads packaged.

**Minimum equipment:** CPR manikin, AED trainer, rhythm strips for post-case ID.

**Instructor notes:** Emphasize high-quality CPR (rate 100–120, full recoil, minimal pauses), early AED, correct pad placement; after ROSC, have learners identify 3 rhythms from binder.

**Reset:** Replace AED pads on backing, close AED, reset manikin chest recoil counter if present, file rhythm strips.

**Case Topic: CPR-AED-ECG Recognition**

**Time:** 20 min.

**Goals:** Identify signs and symptoms, provide initial assessment and management of someone with the need of an AED in cardiac arrest and recognize ECG rhythms.

**Objectives:**

- Determines the scene is safe
- Requests for help
- Introduces self
- Team organization and assignments of roles
- Verbalizes what is happening to person in distress
- Assess for level of alertness
- Verbalizes and assesses
  ○ Airway
  ○ Breathing (look, listen, and feel)
  ○ Circulation
  ○ Disability
  ○ Exposure
- Requests if AED is available (can be earlier)
- Perform high-quality CPR
- Able to place the patient in recovery position
- Acknowledge need for transport to hospital

**Scene:** Gym

**Props:**

- AED CPR Annie
- ECGs in a Binder

**Scenario Set Up:** Patient undergoes a cardiac arrest.

Participants will need to recognize emergency and place AED appropriately.

Participants will need to perform high quality CPR for two cycles

Once CPR is demonstrated, patient regains pulse and case is finished.

At the assessment, will need to identify 3 ECG rhythms.

**Narrative for facilitator to be read to group:**

You’re at the gym on the treadmill, finishing your last mile. The person running next to you starts to slow down abruptly. The person says, “I don’t feel so well,” and then collapses, appearing unresponsive.

Other information that may be asked: (patient unresponsive)

**Name:** Jonah

**Age:** Appears middle-aged

**Medical hx:** No Hx

**Surgery hx:** No Hx

**Medications:** No Hx

**Dosage (if asked):** None

**Allergies:** None

**Physical Exam (if not indicated, normal):**

General: Unresponsive, not moving, not talking
No vital signs. No pulse, not breathing
HEENT: Normocephalic, atraumatic, PERRLA
Circulation: No pulses

**Assessment of Knowledge Questions:**

Identify the following ECG Rhythms:

**TEAM: _________________________________**

**SCORER: _________________________________**

**Table:**

Scoring Rubric (25 points max)	Points Possible	Points Awarded
*Scene Size Up (verbally evaluates scene safety)*	*1*	
*Requests additional EMS assistance*	*1*	
*Introduces self, assignments of roles*	*2*	
*Verbalizes what is happening to person in distress*	*1*	
*Assess for level of alertness*	*1*	
*Verbalizes and assesses* - *Airway* - *Breathing (look, listen, and feel)* - *Circulation* - *Disability* - *Exposure*	*5*	
*Requests if AED is available (can be earlier)*	*3*	
*Perform high quality CPR for two cycles (shows transition between two people)*	*3*	
*CPR continued without any long gaps during transition*	*1*	
*Acknowledge need for transport to hospital*	*2*	
*Identification of ECG Rhythms*	*3*	
*Team organization and style*	*2*	

## Appendix D Debrief Pearls

### Case 1: Allergic Reaction/Anaphylaxis^8^

**Scenario**: A child at a bakery experiences anaphylaxis after eating a cookie with nuts.

**Key Takeaways**:

- **Scene Safety**: Ensure the area is safe.
- **Call for Help**: Request additional assistance (eg, 911).
- **Introduce Self & Assign Roles**: Clear team communication is crucial.
- **Initial Assessment**:
  ○ **Airway**: Check for obstructions.
  ○ **Breathing**: Look, listen, and feel for breath sounds.
  ○ **Circulation**: Monitor pulse and skin color.
  ○ **Disability**: Assess consciousness.
  ○ **Exposure**: Look for rashes or swelling.
- **EpiPen Administration**: Identify need and administer EpiPen; repeat every 5–15 minutes if needed^7,8^
- **Hospital Transport**: Ensure the patient is taken to the hospital.

### Case 2: Opioid Overdose^9^

***Scenario*** *: An unresponsive individual at a subway station, suspected opioid overdose.*

**Key Takeaways**:

- **Scene Safety**: Confirm the scene is safe.
- **Call for Help**: Request additional assistance.
- **Introduce Self & Assign Roles**: Effective team coordination.
- **Initial Assessment**:
  ○ **Airway**: Ensure it is clear.
  ○ **Breathing**: Check for respiration.
  ○ **Circulation**: Look for slow pulse and cyanosis.
  ○ **Disability**: Try to rouse the patient.
  ○ **Exposure**: Examine for other signs.
- **Naloxone Administration**: Administer Naloxone and assess for repeat doses .^9^
- **Hospital Transport**: Ensure transport to the hospital.

### Case 3: Cardiac Arrest (CPR-AED-ECG Recognition)^5,6^

**Scenario**: Cardiac arrest in a gym; participants must perform CPR and use an AED.

**Key Takeaways**:

- **Scene Safety**: Check the scene.
- **Call for Help**: Request EMS assistance.
- **Introduce Self & Assign Roles**: Assign team roles.
- **Initial Assessment**:
  ○ **Airway**: Ensure it is clear.
  ○ **Breathing**: Look, listen, and feel for breath.
  ○ **Circulation**: Check for pulse and signs of life.
- **CPR**: Perform high-quality CPR for two cycles.^5,6^
- **AED Use**: Apply AED and follow prompts.
- **ECG Recognition**: Identify rhythms like VFib, Asystole, and Normal Sinus Rhythm.
- **Hospital Transport**: Arrange for transport to the hospital.

## Appendix E EM Interest Group Case Competition Pre-Survey

### Virtual Escape Room Trainee Evaluation

1. Please state your name ________________________________________
2. What is your email address? (Please enter correctly as this is the modality through which the prizes will be awarded.) ________________________________________
3. What year of medical school are you in?
  ○ M1
  ○ M2
  ○ M3
  ○ M4
4. How did you hear about this event? ________________________________________
5. How confident are you in your ability to respond appropriately to someone with potential anaphylaxis?
  ○ Completely confident
  ○ Fairly confident
  ○ Somewhat confident
  ○ Slightly confident
  ○ Not confident at all
6. How confident are you in your ability to respond appropriately to someone with a potential opioid overdose?
  ○ Completely confident
  ○ Fairly confident
  ○ Somewhat confident
  ○ Slightly confident
  ○ Not confident at all
7. How confident are you in your ability to respond appropriately to someone in cardiac arrest?
  ○ Completely confident
  ○ Fairly confident
  ○ Somewhat confident
  ○ Slightly confident
  ○ Not confident at all
8. How likely are you to respond when a stranger asks if there is anyone with medical training during an emergency?
  ○ Extremely unlikely
  ○ Somewhat unlikely
  ○ Neither likely nor unlikely
  ○ Somewhat likely
  ○ Extremely likely

## Appendix F EM Interest Group Case Competition Post-Survey

### Virtual Escape Room Trainee Evaluation

1. Please state your name ________________________________________
2. What is your email address? (Please enter correctly as this is the modality through which the prizes will be awarded.) ________________________________________
3. What year of medical school are you in?
  ○ M1
  ○ M2
  ○ M3
  ○ M4
4. Was the case competition fun?
  ○ Yes
  ○ No
5. How confident are you in your ability to respond appropriately to someone with potential anaphylaxis?
  ○ Completely confident
  ○ Fairly confident
  ○ Somewhat confident
  ○ Slightly confident
  ○ Not confident at all
6. How confident are you in your ability to respond appropriately to someone with a potential opioid overdose?
  ○ Completely confident
  ○ Fairly confident
  ○ Somewhat confident
  ○ Slightly confident
  ○ Not confident at all
7. How confident are you in your ability to respond appropriately to someone in cardiac arrest?
  ○ Completely confident
  ○ Fairly confident
  ○ Somewhat confident
  ○ Slightly confident
  ○ Not confident at all
8. How likely are you to respond when a stranger asks if there is anyone with medical training during an emergency?
  ○ Extremely unlikely
  ○ Somewhat unlikely
  ○ Neither likely nor unlikely
  ○ Somewhat likely
  ○ Extremely likely
9. How likely will you attend future case competitions?
  ○ Extremely unlikely
  ○ Somewhat unlikely
  ○ Neither likely nor unlikely
  ○ Somewhat likely
  ○ Extremely likely
10. Comments for organizers _____________________________________________________________________________ _____________________________________________________________________________
11. Any special shout outs to our team: _____________________________________________________________________________ _____________________________________________________________________________
